# Alpha-Lipoic Acid Protects Cardiomyocytes against Heat Stroke-Induced Apoptosis and Inflammatory Responses Associated with the Induction of Hsp70 and Activation of Autophagy

**DOI:** 10.1155/2019/8187529

**Published:** 2019-12-03

**Authors:** Hsin-Hsueh Shen, Yu-Shiuan Tseng, Ni-Chun Kuo, Ching-Wen Kung, Sherif Amin, Kwok-Keung Lam, Yen-Mei Lee

**Affiliations:** ^1^Department and Institute of Pharmacology, National Defense Medical Center, Taipei, Taiwan; ^2^Department of Pharmacy Practice, Tri-Service General Hospital, National Defense Medical Center, Taipei, Taiwan; ^3^School of Medicine, National Defense Medical Center, Taipei, Taiwan; ^4^Department of Nursing, Tzu Chi University of Science and Technology, Hualien, Taiwan; ^5^Department of Pharmacology, New York Medical College, New York, USA; ^6^Department of Pharmacology, Taipei Medical University, Taipei, Taiwan; ^7^Department of Anesthesiology, Catholic Mercy Hospital, Hsinchu, Taiwan

## Abstract

Heat stroke (HS) is a life-threatening illness and defined as when body temperature elevates above 40°C accompanied by the systemic inflammatory response syndrome that results in multiple organ dysfunctions. *α*-Lipoic acid (ALA) acts as a cofactor of mitochondrial enzymes and exerts anti-inflammatory and antioxidant properties in a variety of diseases. This study investigates the beneficial effects of ALA on myocardial injury and organ damage caused by experimental HS and further explores its underlying mechanism. Male Wistar rats were exposed to 42°C until their rectal core temperature reached 42.9°C and ALA was pretreared 40 or 80 mg/kg (i.v.) 1.5 h prior to heat exposure. Results showed that HS-induced lethality and hypothermia were significantly alleviated by ALA treatment that also improved plasma levels of CRE, LDH, and CPK and myocardial injury biomarkers myoglobin and troponin. In addition, ALA reduced cardiac superoxide anion formation and protein expression of cleaved caspase 3 caused by HS. Proinflammatory cytokine TNF-*α* and NF-*κ*B pathways were significantly reduced by ALA treatment which may be associated with the upregulation of Hsp70. ALA significantly increased the Atg5-12 complex and LC3B II/LC3B I ratio, whereas the p62 and p-mTOR expression was attenuated in HS rats, indicating the activation of autophagy by ALA. In conclusion, ALA ameliorated the deleterious effects of HS by exerting antioxidative and anti-inflammatory capacities. Induction of Hsp70 and activation of autophagy contribute to the protective effects of ALA in HS-induced myocardial injury.

## 1. Introduction

With climate change, including in some extreme temperatures, such as heat, the growing incidences of heat-related death have been frequently addressed in recent years [1, 2]. Heat stroke (HS) is a life-threatening illness defined as exposure to excessive hyperthermia with body temperature above 40°C and resulting in a systemic inflammatory response syndrome [3]. The pathogenesis of tissue injury in HS is closely mimicked by the mechanism of sepsis [4], which arises from impaired intestinal mucosal barrier and causes translocation of endotoxins [5]. This change allows the secretion of proinflammatory cytokines, including tumor necrosis factor-*α* (TNF-*α*) and interleukin-6 (IL-6), and eventually triggers systemic inflammation [6]. In addition, the bloodstream is switched from the heart and brain to the skin in order to dissipate excessive heat and ultimately develops to hypotension and cardiovascular dysfunction, which are the main causes of HS-related death [7].

Heat shock proteins (Hsps) function as molecular chaperones by facilitating the proper folding of proteins to the native conformation and targeting for lysosomal degradation under stress conditions [8]. Among these Hsps, Hsp70 is one of the most extensively studied proteins induced by an increase in temperature of mammalians. Heat stress causes overexpression of Hsp70 and provides vital protection against HS-induced thermoregulatory deficits, oxidative stress, and the release of proinflammatory cytokines associated with organ dysfunction [9]. Moreover, deletion of the Hsp70 gene in mice debilitated calcium conduction and contractility of cardiomyocyte [10]. Thus, the induction of Hsp70 may be a potential strategy for the treatment of HS-induced cardiomyocyte injury.

Autophagy is a cellular process of lysosomal degradation and plays an important role in the degradation of damaged organelles and misfolded proteins as well as elimination of intracellular pathogens [11]. Specifically, autophagy emerged as a major regulator of cardiac homeostasis and is widely implicated in heart disease, including cardiomyopathy, heart failure, and ischemia-reperfusion injury [12]. The microtubule-associated protein light chain 3 B (LC3B) is correlated with the extent of autophagosome formation during autophagy induction. This autophagic process is considered as a cytoprotective mechanism in response to oxidative stress, infectious diseases, and adaptation to stress [13]. However, investigation of the role of autophagy in the myocardium under HS injury is crucial but relevant studies thus far are still very limited.

Alpha-lipoic acid (ALA) is an organo-sulphated compound derived from octanoic acid and considered as one of the most potent cellular antioxidants [14]. ALA can be reduced to dihydrolipoic acid and serves as a strong antioxidant for reactive oxygen species (ROS), as a scavenger, and provision of glutathione [15]. The therapeutic potential of ALA has been widely demonstrated in a variety of oxidative stress-related diseases, including neurodegeneration, diabetic neuropathies, and cardiovascular diseases [16, 17]. Previously, we demonstrated that ALA ameliorates multiple organ injuries in endotoxemic rats through its antioxidant and anti-inflammatory capacities [18]. Specifically, ALA exerts anti-inflammatory effects via the inhibition of nuclear factor-*κ*B (NF-*κ*B) in various biological models [19, 20]. However, the impact of ALA on systemic inflammatory responses and cardiomyocyte injuries under HS remains to be elucidated. Thus, the objective of the present study is to investigate the effects of ALA on an animal model of HS-induced injury of organs and further explore whether Hsp70 and autophagy are associated with the protective effects.

## 2. Materials and Methods

### 2.1. Experimental Animals

All procedures for animal handling were in accordance with the *Guide for the Care and Use of Laboratory Animals published by the US National Institutes of Health* (NIH Publication No. 85-23, revised in 1996). Male Wistar rats (8 weeks old, 300–350 g) were obtained from BioLASCO Co., Ltd., Taiwan. This study was approved by the Institutional Animal Care and Use Committee of the National Defense Medical Center, Taiwan (Number of Permission: IACUC-14-160).

### 2.2. Experimental Groups

**Figure 1:**
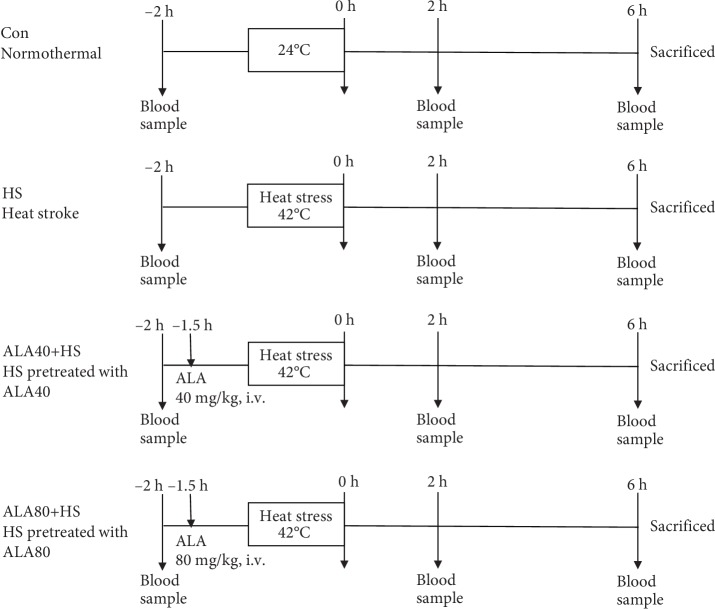
Experimental protocol. Con: normothermal control; HS: heat stroke; ALA40+HS: *α*-lipoic acid (40 mg/kg, i.p.) was administered 90 min before heat stroke induction; ALA80+HS: *α*-lipoic acid (80 mg/kg, i.p.) was administered 1.5 h before heat stroke induction.

After anesthesia (urethane 1.2 g/kg, i.p.) and left femoral arterial and venous cannulation, the rats were randomized into four groups as indicated in Figure 1: (I) normothermal control group (Con): rats were arranged in a heating chamber at room temperature (24°C) for 1 h and maintained throughout the entire experiment (*N* = 5); (II) HS group: rats were placed in a heating chamber (42°C) for 40 min until the rectal core temperature (Tco) elevated to 42°C to induce HS, as previously described [21] (*N* = 19); (III) ALA40+HS group: HS rats were pretreated with ALA 40 mg/kg (i.p.) (Sigma-Aldrich, St. Louis, MO, USA) at 1.5 h prior to heat stress (*N* = 12); (IV) ALA80+HS group: rats were pretreated with ALA 80 mg/kg (i.p.) at 1.5 h prior to HS (*N* = 7). After being removed out of the heating chamber, the survival rate was monitored for 6 h and animals were then sacrificed by the injection of pentobarbital (50 mg/kg, i.v.). Blood and heart samples were collected immediately for further analysis.

### 2.3. Biochemical Assays

Whole blood (0.5 mL) was collected into tubes containing sodium citrate and centrifuged (10,000 g for 3 min) to prepare plasma. Plasma levels of lactate dehydrogenase (LDH) (cell toxicity index), glutamic pyruvic transaminase (GPT) (hepatic function index), creatinine (CRE) (renal function index), and creatine phosphokinase (CPK) and myoglobin (rhabdomyolysis markers) were measured at baseline (-2), 2, and 6 h after HS, as determined using a Fuji DRI-CHEM 3030 analyzer (Fuji Photo Film, Tokyo, Japan). Plasma cardiac troponin I was determined using luminescence immunoassay (Automated Chemiluminescence System, Bayer, Co. NY, USA) with an assay kit (Abbott Diagnostics, New Jersey, USA) according to the manufacturer's protocol.

### 2.4. Determination of Superoxide Formation in the Myocardium by Chemiluminescence

The myocardium was derived from the left ventricular apex and cut into 3 × 3 mm in size. Superoxide anion production is determined by lucigenin-derived chemiluminescence. Briefly, samples were placed into a 96-well plate filled with 200 *μ*L modified Krebs-HEPES solution and placed in a microplate luminometer (Hidex, Microplate Luminometer, Finland). After recording background counts, the myocardium sample was added to each well and incubated with 50 *μ*L lucigenin (125 *μ*M) for 1 min. Counts were then recorded for each well, and the respective background was subtracted. After recording, the myocardium was dried in a drying cabinet for 24 h. These results were expressed as counts per second (cps) per milligram dry weight of tissues, as previously described [22].

### 2.5. Western Blot Analysis

Detection of the protein expression in the ventricular myocardium by Western blot was performed as described previously [23]. The protein concentration of the supernatant was measured by the BCA kit (Thermo Scientific, Waltham, MA, USA). Twenty micrograms of protein extract was separated on 10% sodium dodecyl sulfate-polyacrylamide gels and transferred to a nitrocellulose membrane. After blocking in 5% bovine serum albumin for 1 hour, blots were then incubated overnight at 4°C with the following primary antibodies: anti-NF-*κ*B p-p65, anti-p65, anti-cleaved caspase 3, anti-LC3B, anti-Atg5-12, anti-p62, anti-p-mTOR, and anti-mTOR (all 1 : 1000, Cell Signaling Technology, Danvers, MA, USA); anti-TNF-*α* (1 : 1000, Abcam, Cambridge, MA, USA); anti-Hsp70 (1 : 1000, Enzo Life Sciences, Farmingdale, NY, USA); anti-*α*-actin (1 : 5000, GeneTex, Irvine, CA, USA). The membranes were then probed with horseradish peroxidase-conjugated secondary antibodies. Proteins were visualized using enhanced chemiluminescence reagents (Bio-Rad, Hercules, CA, USA) and quantified by densitometry of the blots using ImageJ software.

### 2.6. Statistical Analysis

The data are presented as the means ± standard errors of the mean (SEM). Statistical analysis was performed with one-way analysis of variance (ANOVA) followed by the Newman–Keuls *post hoc* multiple comparison methods. The chi-square test followed by the Fisher's exact test was used for comparison of the survival distributions between groups of rats. The Bonferroni test was used to correct multiple comparison of survival distribution. The Kaplan-Meier method was used to calculate the survival rate, and the log-rank test was used to test differences between groups. Differences were considered statistically significant at *P* < 0.05.

## 3. Results

### 3.1. Effects of ALA on Survival Rate and Core Temperature (Tco) in Heat Stroke Rats

**Figure 2:**
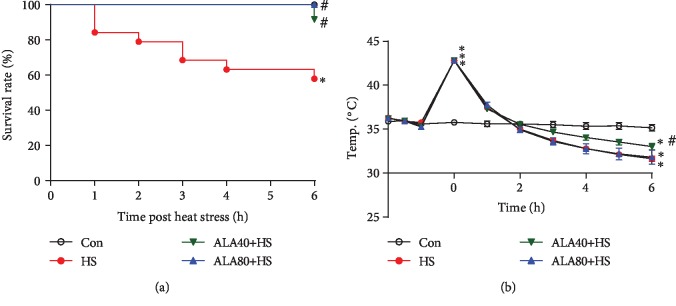
Effects of pretreatment with *α*-lipoic acid (ALA) on (a) survival rate and (b) core temperature in heat stroke rats. Data are expressed as mean ± SEM. ^∗^*P* < 0.05 versus Con; ^#^*P* < 0.05 versus HS.

Six hours after HS onset, the survival rate decreased to 57.9% (11/19 animals) and was significantly reduced as compared to the Con group (100%, 5/5 animals) (Figure 2(a)). In the ALA40+HS and ALA80+HS groups, the survival rate was 91.7% (11/12 animals) and 100% (7/7 animals), respectively, which was significantly higher than that of the HS group (*P* < 0.05).  Figure 2(b) shows that the rats exposed to heat stress exhibited significant decreases in core temperature (Tco) at 6 h when compared to the Con group. However, the development of the deficits in thermoregulation (hypothermia) was prevented by ALA 40 mg/kg administration in HS rats.

### 3.2. Effects of ALA on HS-Induced Organ Damage in Rats

**Figure 3:**
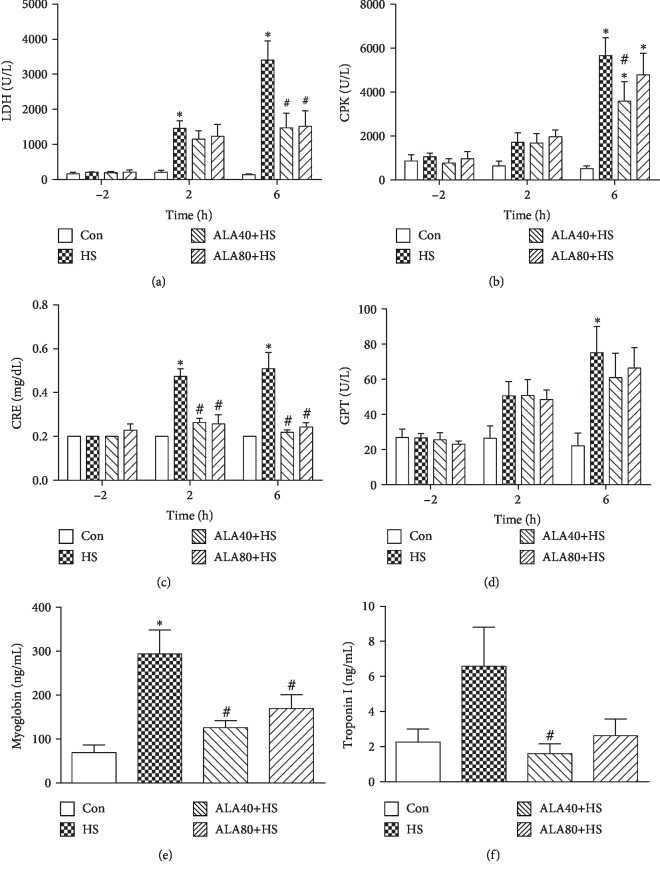
Effects of pretreatment with *α*-lipoic acid (ALA) on plasma levels of (a) lactate dehydrogenase (LDH), (b) creatine phosphokinase (CPK), (c) creatinine (CRE), (d) glutamic pyruvate transaminase (GPT), (e) myoglobin, and (f) troponin I in heat stroke rats. Data are expressed as mean ± SEM (*n* = 5-11). ^∗^*P* < 0.05 versus Con; ^#^*P* < 0.05 versus HS.

The baseline levels of LDH, CPK, CRE, GPT, and myoglobin were not significantly different among groups. After HS initiation, plasma levels of LDH, CPK, CRE, GPT, and myoglobin increased progressively during the span of 2 h in the HS group, and all were significantly higher than those of the Con group at 6 h (*P* < 0.05). The HS-induced elevation of plasma levels of LDH and CRE was significantly attenuated in the ALA40+HS and ALA80+HS groups (Figures 3(a) and 3(c)). ALA 40 mg/kg treatment in HS rats also prevented the increase of plasma CPK values at 6 h (Figure 3(b)). However, no significant difference in GPT levels was observed by ALA 40 and 80 mg/kg pretreatment in HS rats (Figure 3(d)).

### 3.3. Effects of ALA on HS-Induced Cardiomyocyte Injuries in Rats

Serum myoglobin and troponin I are implicated as biomarkers for myocardial injury because of the continuous release of myofilament components from the injured cardiac muscle [24]. As shown in Figures 3(e) and 3(f), HS induced notably an increase in plasma myoglobin and troponin I levels as compared to the Con group. Meanwhile, ALA treatment significantly decreased the myoglobin and troponin I concentrations when compared with those of the HS group.

### 3.4. Effects of ALA on Cardiac Apoptosis-Related Protein Cleaved Caspase 3 Expression in Heat Stroke Rats

**Figure 4:**
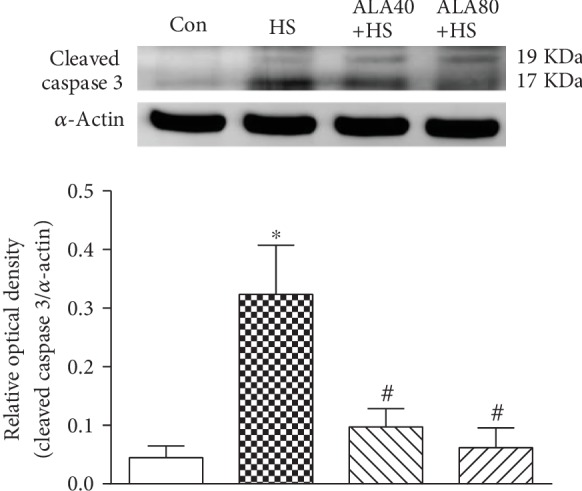
Effects of pretreatment with *α*-lipoic acid (ALA) on cleaved caspase 3 protein expression in the myocardium of heat stroke rats. Data are expressed as mean ± SEM (*n* = 5-8). ^∗^*P* < 0.05 versus Con; ^#^*P* < 0.05 versus HS.

Six hours after HS induction, the cleaved caspase 3 protein expression in the HS group was significantly higher than that in the Con group (Figure 4) (*P* < 0.05). Pretreatment with ALA 40 or 80 mg/kg significantly attenuated the cleaved caspase 3 expression induced by HS. The results indicate that the protective effect of ALA on HS is associated with the attenuation of cardiac apoptosis.

### 3.5. Effects of ALA on Superoxide Anion Formation in the Myocardium of Heat Stroke Rats

**Figure 5:**
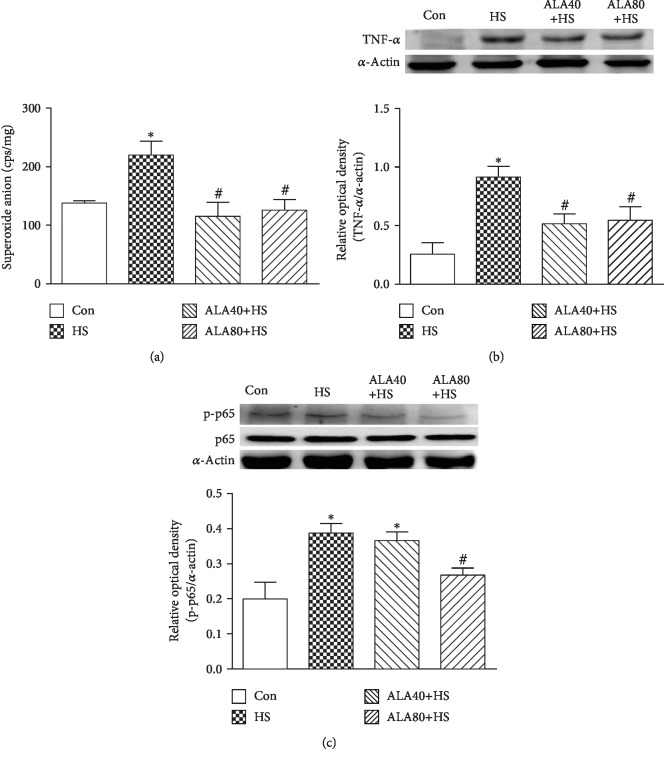
Effects of pretreatment with *α*-lipoic acid (ALA) on (a) superoxide anion formation and (b) TNF-*α* and (c) NF-*κ*B p-P65 protein expression in the myocardium of heat stroke rats. Data are expressed as mean ± SEM (*n* = 5-8). ^∗^*P* < 0.05 versus Con; ^#^*P* < 0.05 versus HS.

It is reported that ALA provides antioxidant activity through ROS scavenging and its ability to regenerate antioxidants [15]. As shown in Figure 5(a), the levels of the superoxide anion formation in the ventricular myocardium of the HS group were significantly higher than those of the Con group (Con, 137.9 ± 3.6 cps/mg tissue; HS, 220.0 ± 23.5 cps/mg tissue). The increase in the superoxide anion formation in the HS group was significantly suppressed in the ALA40+HS and ALA80+HS groups (ALA40+HS, 114.9 ± 24.0 cps/mg tissue; ALA80+HS, 125.7 ± 18.3 cps/mg tissue) (*P* < 0.05).

### 3.6. Effects of ALA on Cardiac Inflammation-Related Protein TNF-*α* and NF-*κ*B p-p65 Expression in Heat Stroke Rats

The excessive production of superoxide anions may trigger the accumulation of inflammatory mediators [25].  Figure 5(b) shows that the HS challenge resulted in increased protein levels of TNF-*α* in the myocardium at 6 h, which was significantly reduced in the ALA 40+HS and ALA80+HS groups. In addition, the activation of the NF-*κ*B pathway plays a key role in mediating the inflammatory responses [26], and its activity was determined by the phosphorylated p65 (p-p65) subunit. As shown in Figure 5(b), NF-*κ*B p-p65 protein expression was significantly increased in the HS group when compared with the Con group. The HS-induced increase of NF-*κ*B p-p65 protein expression was significantly diminished by ALA 80 mg/kg administration (Figure 5(c)).

### 3.7. Effects of ALA on Cardiac Hsp70 Protein Expression in Heat Stroke Rats

**Figure 6:**
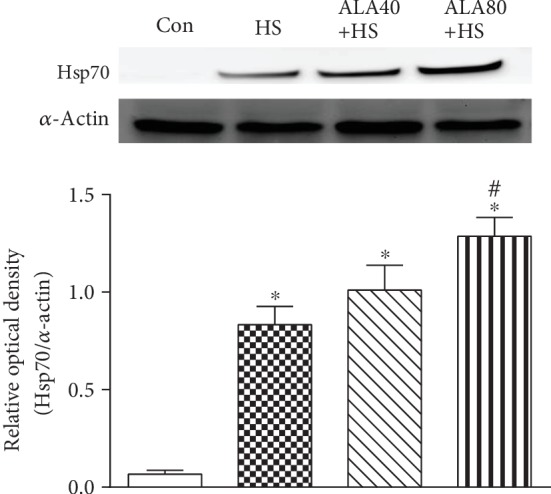
Effects of pretreatment with *α*-lipoic acid (ALA) on Hsp70 protein expression in the myocardium of heat stroke rats. Data are expressed as mean ± SEM (*n* = 5-8). ^∗^*P* < 0.05 versus Con; ^#^*P* < 0.05 versus HS.

Hsp70 has been reported to activate the autophagic process in response to heat stress and protects against HS-induced organ damage [21, 27]. As shown in Figure 6, Hsp70 protein expression in the HS group was significantly higher than that in the Con group in the hearts. Pretreatment with ALA 80 mg/kg (ALA80+HS group) significantly enhanced the upregulation of HS-induced Hsp70 protein expression.

### 3.8. Effects of ALA on the Expression of Cardiac Autophagy Marker Proteins in Heat Stroke Rats

**Figure 7:**
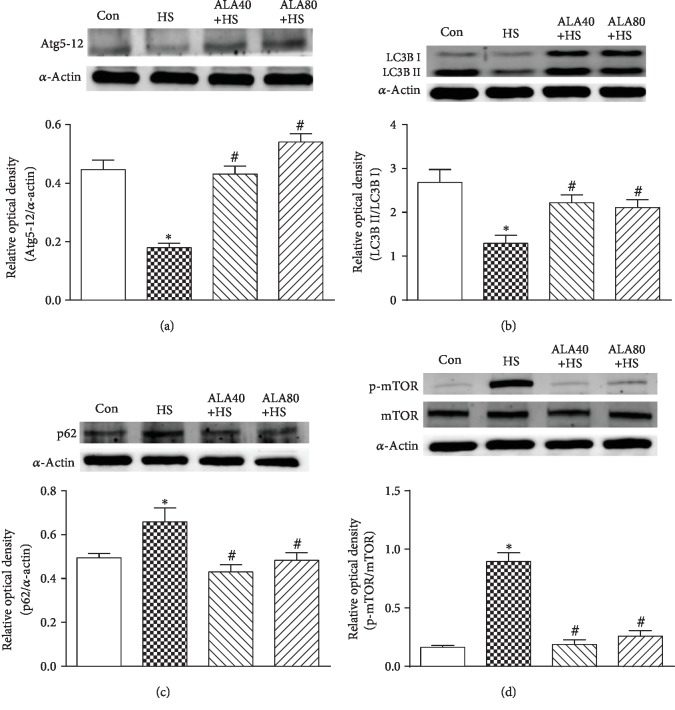
Effects of pretreatment with *α*-lipoic acid (ALA) on (a) Atg5-12, (b) LC3B, (c) p62, and (d) mTOR protein expression in the myocardium of heat stroke rats. Data are expressed as mean ± SEM (*n* = 5-8). ^∗^*P* < 0.05 versus Con; ^#^*P* < 0.05 versus HS.

To further elucidate the relationship between the protective effect of ALA and autophagy, we evaluated the expression levels of autophagy-related protein 5-12 (Atg5-12), LC3B, p62/SQSTM1 (p62), and the phosphorylated mammalian target of rapamycin (p-mTOR), which have been recognized as autophagy-associated markers [11]. Six hours after HS, the levels of the Atg5-12 complex were significantly decreased in the HS group as compared with the Con group (Figure 7(a)). Pretreatment with ALA 40 and 80 mg/kg showed significantly higher levels of Atg5-12 complex than those of the Con group. As shown in Figure 7(b), the ratio of LC3B II/LC3B I level in the HS group was lower than that in the Con group (Figure 7(b)). Pretreatment with ALA 40 and 80 mg/kg increased significantly the ratio of LC3B II/LC3B I protein expression in HS-stimulated cardiac tissues.

Adaptor protein p62 carries the target substrate for degradation into the autophagosome, and its amount inversely correlates with autophagic degradation [28]. As shown in Figure 7(c), the protein expression of p62 in the HS group was significantly higher than that in the Con group, indicating that HS caused failure on the autophagosome degradation. However, pretreatment with ALA 40 and 80 mg/kg reversed the accumulation of p62 in the HS group.

The protein kinase mammalian target of rapamycin (mTOR) signaling also negatively regulates autophagy, i.e., the phosphorylation and activation of mTOR are suppressed while autophagy is induced [29]. The result in Figure 7(d) shows that HS markedly induced the phosphorylation of mTOR (at the serine 2448 site); concurrently, both pretreatment with ALA 40 and 80 mg/kg reversed the increase of p-mTOR expression after HS.

## 4. Discussion

In the present study, we demonstrated that pretreatment with ALA reduced mortality and hypothermia and alleviated organ damage caused by heat stroke. The decrease in myoglobin and troponin I of HS rats may be a consequence of the protective effects of ALA in the cardiovascular system. In addition, increased apoptotic signaling associated with oxidative stress following HS induction was mitigated by ALA. Attenuation of inflammatory cytokine TNF-*α* levels and inhibition of the activation of the NF-*κ*B signaling pathway are contributed to the protective effects of ALA. The underlying mechanism is associated with the induction of Hsp70 and preservation of autophagy by ALA under heat stress.

Elevation of body core temperature results in the increase of skin blood flow and the reduction of splanchnic blood flow [30], leading to hypoxia and oxidative stress in the gut, which causes the increase of gut epithelial permeability, and the leakage of endotoxin from the intestine to circulation. The systemic inflammatory response syndrome is elicited by endotoxin and ensues following damage to the gut and other organs. Multiorgan system failure is the final cause of mortality. Increasing evidence supports the findings that cardiovascular dysfunction is the most common complication among HS patients, manifested as cardiomyocyte apoptosis, ischemic heart disease, and heart failure [31]. The excessive production of ROS by heat stress may trigger oxidative stress and accumulation of inflammatory mediators, ultimately leading to cell apoptosis or necrosis [32]. Manipulation of antioxidant capacities may alleviate heat-mediated apoptosis and help to increase cellular defense. Several lines of evidence demonstrated the cardioprotective effects of ALA against ischemia-reperfusion and LPS-induced cardiac injury through its antioxidant property [33, 34]. In our experimental model of HS, pretreatment with ALA effectively reduced the superoxide anion formation in the heart, demonstrating its antioxidant property, and can be considered as a potential intervention strategy for HS-associated oxidative damage.

Infection and local tissue injury generate the release of proinflammatory cytokines including TNF-*α* and IL-6, which contribute to increased systemic inflammatory responses. TNF-*α* induces a wide range of biological effects including cell differentiation, apoptosis, and multiple proinflammatory effects, which trigger the activation of the NF-*κ*B signaling pathway [35]. ALA has been shown to suppress NF-*κ*B activation through direct ROS scavenging [19] or even independent of its antioxidant function [36]. In accordance with these studies, the antioxidant capacity of ALA is contributed to its anti-inflammatory effect in the present animal model of heat stroke.

Heat shock response and autophagy represent two distinct systems to maintain cellular protein quality and complement each other under stress conditions. Autophagy has been implicated in a variety of heart diseases, including cardiomyopathy and ischemia/reperfusion injury; however, its role in the cardioprotective effect of HS rats remains controversial [37]. Heat stress could result in protein aggregation and promote a dysfunctional intracellular environment [38]. Insufficient autophagy or partial degradation of damaged organelles causes the generation of ROS and loss of lysosomal function, resulting in cardiac remodeling and cell death [12, 39]. In addition, inhibition of autophagy was associated with the development of cardiac hypertrophy in response to the cardiac-specific deletion of Atg5 in mouse cardiomyocytes [40]. Thus, targeting autophagy activation may provide a potential therapeutic approach for heat stress-induced cardiovascular dysfunction. During autophagy, the conversion of cytosolic LC3B I to LC3B II is required for autophagic membrane recruitment and can be regarded as the indicator of autophagy induction. Elongation of the nascent autophagosomal membrane is regulated by Atg5-12 and the LC3B ubiquitin-like conjugation system. It has been shown that activation of autophagy is one of the protective mechanisms against neurodegeneration in HS [41]. Heat stress increased accumulation of unfolded proteins and resulted in autophagy induction. The autophagy machinery targets intracellular pathogens for degradation, modulates inflammation, and participates in adaptive immune responses [42]. Furthermore, cells that are unable to cope with the stress responses by autophagy may be ultimately subject to apoptosis. It is demonstrated that inhibition of autophagy accelerated apoptosis and disturbed immunity as well as increased ROS production [43], suggesting that autophagy plays an important role in the inhibition of apoptosis and inflammation. The causal relationship of ALA and autophagy was demonstrated in recent literature that ALA activated autophagy and reduced NF-*κ*B signaling as well as apoptosis to exert the protective effects in the myocardium of septic rats [44]. However, these effects were significantly eliminated by the administration of autophagy inhibitor 3-methyladenine (3-MA), suggesting that enhancement of autophagy played a critical role in the protective effect of ALA in alleviation of acute inflammation. In this study, ALA pretreatment increased autophagy and preserved the capacity of autophagosome formation in response to heat stress in the cardiomyocytes, resulting in promoted degradation of misfolded protein aggregates.

Heat shock proteins induced by heat stress or oxidative damage are functioning as molecular chaperones and exert cell cycle regulatory and antiapoptotic activities [45]. A hallmark in the endogenous protective mechanism against HS-induced oxidative injury is the upregulation of Hsp70, which acts as a chaperone in facilitating the folding and refolding of proteins for cellular homeostasis [9]. Heart preconditioning induces the Hsp70 expression which prevented cardiomyocytes from HS damage and reduced apoptosis [46]. Evidence has also been shown that Hsp70 preserved redox balance via increasing the glutathione-related enzymes [47] and preinduction of Hsp70 improved cardiac mechanical efficiency and hypotension [48], which might be mediated through chaperone-assisted selective autophagy [49]. In addition, induction of Hsp70 has been shown to exert the protective effect by inhibition of apoptosis-related proteins in cardiomyocytes, including apoptosis-inducing factor and eukaryotic translation elongation factor 2, thus alleviating apoptosis [50]. In this *in vivo* study, increases in Hsp70 protein expression after a high dose of ALA (80 mg/kg) administration were in accordance with a previous *in vitro* observation that ALA increased Hsp70 mRNA and its protein expression with the end to preserve the intestinal epithelial integrity under heat stress in Caco-2 cells [51]. Our previous study also indicated that upregulation of Hsp70 is involved in the activation of autophagy, resulting in protection against HS-induced organ damage [21]. In the present study, ALA was able to trigger the activation of autophagy and induction of Hsp70. These two functionally distinct systems cooperate in maintaining cellular homeostasis by promoting appropriate folding of unfolded proteins or removing damaged proteins to facilitate in coping with the cellular stress.

In conclusion, ALA possesses antioxidant, anti-inflammatory, and antiapoptotic effects to prevent HS-induced cardiomyocyte dysfunction. Induction of Hsp70 and activation of autophagy may contribute to the beneficial effects of ALA against heat stroke.

## Data Availability

The raw/processed data used to support the findings of this study are available from the corresponding author upon request.
